# RETRACTION: Korean Red Ginseng Suppresses Metastasis of Human Hepatoma SK-Hep1 Cells by Inhibiting Matrix Metalloproteinase-2/-9 and Urokinase Plasminogen Activator

**DOI:** 10.1155/ecam/9839547

**Published:** 2025-05-21

**Authors:** Evidence-Based Complementary and Alternative Medicine

RETRACTION: Y.-L. Ho, K.-C. Li, W. Chao, Y.-S. Chang, and G.-J. Huang, “Korean Red Ginseng Suppresses Metastasis of Human Hepatoma SK-Hep1 Cells by Inhibiting Matrix Metalloproteinase-2/-9 and Urokinase Plasminogen Activator,” *Evidence-Based Complementary and Alternative Medicine* 2012 (2012), https://doi.org/10.1155/2012/965846.

The above article, published online on 17 April 2012 in Wiley Online Library (https://wileyonlinelibrary.com), has been retracted by John Wiley & Sons Ltd.

The retraction has been agreed following the investigation of concerns raised by *Actinopolyspora biskrensis* and *Penicillium citreoviride* on PubPeer [1]. The investigation has identified similarities across 3 panels in Figure 4b: control and 100 and 200 µg/mL. As a result of the investigation, the data and conclusions of this article are considered unreliable.

The authors were informed of the decision to retract the article but did not provide a response.
